# New nurses’ practice environment, job stress, and patient safety attitudes: a cross-sectional study based on the job demands-resources model

**DOI:** 10.1186/s12912-024-02135-0

**Published:** 2024-07-12

**Authors:** Xin Wang, Ming Liu, Tao Xu, Kangyue Wang, Liebin Huang, Xiancui Zhang

**Affiliations:** 1 Faculty of Health Sciences and Sports, Macao Polytechnic University, Macao, China; 2 Peking University Health Science Center - Macao Polytechnic University Nursing Academy, Macao Polytechnic University, Macao, China; 3 School of Nursing, Hangzhou Normal University, Hangzhou, Zhejiang China; 4 Health Management Center, The First Affiliated Hospital of Wannan Medical College, Wuhu, Anhui China

**Keywords:** Job demands–resources, Job stress, New nurses, Patient safety attitudes, Practice environment

## Abstract

**Background:**

Patient safety is paramount for all healthcare agencies. Health professionals' lack of patient safety competencies threaten patients’ lives, and increase patients, families, hospitals, and social burdens. The new nurse-related patient safety issues have particularly attracted much attention. The aim of this study was to examine the impacts of practice environment and job stress on new nurses' patient safety attitudes by employing the job demands–resources model.

**Methods:**

The study used a cross-sectional structural equation modeling (SEM). A convenience sample of 370 new nurses was recruited from seven tertiary hospitals in Anhui province, China, from April 2022 to August 2022. Data were collected using self-report questionnaires including the Chinese version of the Nurse Job Stressors Scale, the Practice Environment Scale, and the Safety Attitudes Questionnaire.

**Results:**

New nurses' patient safety attitudes scores were moderate (126.99 ± 14.39). Practice environment had a significant direct effect on job stress (β = -0.337, t = 6.120), patient safety attitudes (β = 0.604, t = 13.766), practice environment had an indirect effect on patient safety attitudes through job stress (β = 0.066, t = 3.690), and the indirect effect accounted for 9.9% of the total effect. This model was able to explain 48.3% of patient safety attitudes with moderate prediction accuracy.

**Conclusions:**

This study emphasizes the importance of improving new nurses' attitudes toward patient safety. Hospital administrators should develop policies and strategies to address job characteristics, and establish a favorable work environment to reduce new nurses' job stress as well as to improve patient safety.

## Background

Patient safety is a fundamental ethical and moral principle grounded in the healthcare ethos of "First, do no harm!". The World Health Organization (WHO) emphasizes the importance of patient safety as the foundation of healthcare delivery in all contexts [1]. Nonetheless, 10% of patients suffer harm from healthcare services, with over three million avoidable deaths occurring annually as a result of substandard care [2]. Research has shown that despite systematic theoretical learning and clinical placements, new nurses continue to be at high risk of care errors [3].

New nurses are usually new graduates or new entrants to the profession, which are defined as nurses working within 2 years of graduation [4]. As per Benner's theory, nurses possess varying degrees of theoretical and practical knowledge at different stages of their vocation [5]. However, there is a more significant gap between new nurses' theoretical knowledge and actual clinical care [6], and the limited experience they are exposed to healthcare systems leads to difficulties and transition pressures for new nurses entering clinical practice [7]. It has also been established that a new nurse transition from student to clinical nurse is accompanied by a degree of shock [8]. These shocks will lead to significant job-related stress and burnout for novice nurses, resulting in a decline in their work quality. In summary, these factors make new nurses susceptible to potential errors and challenges to patient safety. In addition, to address safety concerns, a shift towards systems-based investigation is necessary.

Attainment of patient safe practice is contingent on various factors, including systemic and organizational facets, technological systems, human conduct and behaviors, patient-related dynamics, and external determinants (such as the absence of policies, and inconsistent regulations) [1]. In this context, it is important to explore the influence of job characteristics on the attitudes of newly qualified nurses toward patient safety and also to identify the problems of new nurses' adaptation to the healthcare system. Therefore, this study uses the Job Demands-Resources (JD-R) model to examine how different job characteristics influence new nurses' attitudes toward patient safety.

### Theoretical framework

The study is based on the JD-R model [9], which groups job characteristics into job demands and resources. This model has been commonly applied in nursing practice and explores the role of work participation in nursing practice [10]. According to this theoretical model, various job demands and resources can interact and jointly affect organizational outcomes. Patient safety attitudes are a crucial organizational outcome in clinical settings [11]. In light of this, we employed this model to investigate the effects of various work factors of new nurses on patient safety.

**Fig. 1:**
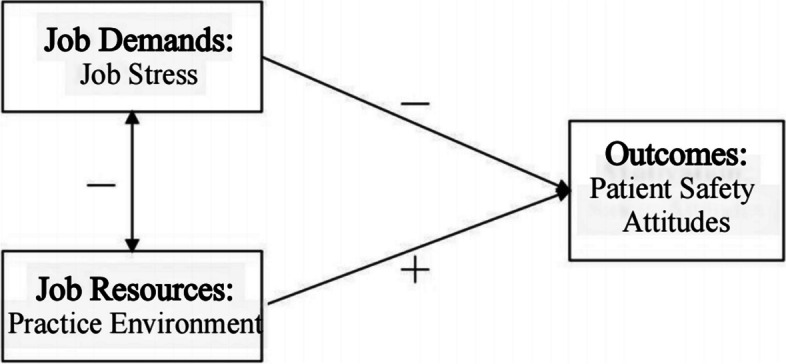
Job demands-resources model

Job demands were defined as: "physical, psychological, social or organizational aspects of the job that require sustained physical and/or psychological (cognitive and emotional) effort or skills and are therefore associated with certain physiological and/or psychological costs ([9], p312])." Research has shown that job-related stress in nursing is associated with adverse effects on both physical [12] and mental [13] health of nurses. Furthermore, increased stress levels lead to lower quality of care and negative nursing outcomes [14]. In this study, new nurses' job stress is considered the job demand. Job resources were defined as: "functional in achieving work goals; reduce job demands and the associated physiological and psychological costs; or stimulate personal growth, learning, and development ([9], p312])." High levels of job resources will significantly improve motivation and organizational outcomes, and we use a practice environment as a job resource because a positive practice environment reduces work-related stress [15] and improves nursing outcomes. In this study, we proposed a theoretical model based on the JD-R and analyzed the links between the practice environment, job stress, and patient safety attitudes among new nurses (Fig. 1).

#### Practice environment

The practice environment can be defined as a system and support structure that develops and implements policies and protocols within which one works and interacts to ensure quality care between patients and family members, nurses and other professionals, support staff, and educators [16]. When new nurses are transitioned from student to a nurse, the impact of the work environment cannot be ignored, and it has a significant effect on retention intentions, physical and mental health, and nursing outcomes [17, 18]. A suboptimal practice environment could result in unsatisfactory operational results, as well as unfavorable patient satisfaction and safety outcomes [19]. Thus, our study examines the impact of the practice environments, which we have utilized as an independent variable, on new nurses' job-related stress and attitudes towards patient safety.

#### Job stress

Job stress is an individual's psychological and physiological stress response in the work environment due to a mismatch between job requirements and personal abilities, resources, or needs [20]. The JD-R model considers [9] that job demands (job stress) reduce the impact of work resources (practice environment) on organizational outcomes (patient safety attitudes). Several studies have shown that new nurses experience higher levels of job stress [21, 22], and heavy workloads have been identified as the main source of stress [23]. An American study found that new nurses reported higher levels of stress due to a lack of professional nursing competence, fear of making mistakes, being in unfamiliar situations, and performing new tasks [24]. Besides, stressful jobs can affect nurses' mental health and well-being and reduce their productivity, performance and quality of patient care [25]. Consequently, we proposed that job stress may act as a mediating factor.

#### Patient safety attitudes

Patient safety is defined as actions undertaken to prevent and eliminate damages that may affect patients and their families during the provision of health care by healthcare professionals [26]. Patient safety attitudes are regarded as the ideological basis of medical safety, the basis for action and intrinsic motivation, which can regulate the safety behavior of medical staff, organize and coordinate safety management, and bring the healthcare organization into a benign state of orderly development [27]. Cultivating a positive patient safety culture is crucial in enhancing patient safety [28]. New nurses with limited clinical experience may be riskier to patient safety issues when providing care [29]. Therefore, it is significant to investigate the patient safety attitudes of new nurses.

#### Hypothesized model

Based on the above theoretical framework, we hypothesized that the clinical practice environment directly affects patient safety attitudes and indirectly affects patient safety attitudes through new nurses' job stress. The aims of this study were: (1) to investigate new nurses' patient safety attitudes; (2) to explore the relationship between practice environment, job stress and patient safety attitudes; (3) to test the mediating role of job stress in the relationship between practice environment and patient safety attitudes.

## Methods

### Study design

This study used a cross-sectional design and followed the STROBE guideline for cross-sectional studies [30].

### Participants

Participants were selected from seven general tertiary hospitals across six cities in Anhui, China. Inclusion criteria were: (1) possession of a Chinese nursing certificate; (2) work experience within a range of 1 to 2 years, and (3) currently full-time working nurses. The study excluded individuals: (1) not engaged in clinical nursing during the research period, such as those on maternity or sick leave; and (2) refuse to sign informed consent form.

### Study sample

Klien [31] recommended a minimum sample size of 200 for stable estimates when testing SEM. According to the SEM estimation parameters calculation method by Reykov and Marcoulides [32], the model is expected to have 35 estimated parameters. Empirically, the sample size is calculated as ten times the estimated parameters [33], resulting in a sample size of 350. Considering a possible 10% non-response rate and potential invalid questionnaire, we used a convenience sampling method to recruit a total of 400 new nurses.

### Data collection

The data were collected from April 2022 to August 2022. The trained investigators traveled to the hospitals where the participants worked. Before distributing the questionnaires, the participants were informed of the study's objective, process, and ethical principles followed by signing the informed consent. Four hundred questionnaires were distributed, 390 were collected, and 20 invalid questionnaires were excluded due to incompletion. A total of 370 participants completed the questionnaires with a 92.5% response rate.

### Measures

#### Socio-demographic characteristics

The sociodemographic variables included age, gender, education level, months of work, number of rotating departments, work of units, and locales.

#### Practice environment

The Practice Environment Scale (PES) was designed by Lake [34], translated into Chinese and validated by Li [35]. The scale is a 4-point Likert scale from 1 (completely disagree) to 4 (completely agree) and consists of 31 items within 5 domains: nurse participation in hospital affairs (9 items), nursing foundations for quality of care (10 items), nurse manager ability, leadership, and support of nurses (5 items), staffing and resource adequacy (4 items), and collegial nurse–physician relations (3 items). Total scores range from 31 to 124; a higher score indicates a better practice environment. The Cronbach's α of the entire scale is 0.970, and 0.822 to 0.927 for subdimensions.

#### Job stress

Based on Grey-Toft's [36] and Wheeler's [37] Nurse Job Stress Scale, Chinese scholar Li [38] developed and modified the scale to assess the job stress status of clinical nurses. It is a 4-point Likert scale from 1 (never experienced) to 4 (experienced almost every day) and consists of 5 domains, 35 items: nurse profession and work (7 items), workload and time allocation (5 items), work environment and resources (3 items), patient care (11 items), and management and dressing (9 items). Total scores range from 35 to 140; higher scores indicate higher job stress. The Cronbach's α of the entire scale is 0.961, and 0.844 to 0.944 for subdimensions.

#### Patient safety attitudes

The Safety Attitudes Questionnaire (SAQ) was designed by Sexton JB [39] to assess medical staff's views on safety attitudes in their departments and medical institutions. The Chinese version of the SAQ was tested for cross-cultural adaptation and psychometric properties were validated by Guo [40]. The scale is a 5-point Likert scale from 1 (strongly disagree) to 5 (strongly agree) and contains 6 domains, 31 items: teamwork climate (6 items), safety climate (7 items), perceptions of management (4 items), job satisfaction (5 items), working conditions (5 items) and stress recognition (4 items). Items 6 and 13 are reverse-scored, total scores range from 31 to 155; higher scores indicate better safety attitudes. The Cronbach's α of the entire scale is 0.911, and 0.682 to 0.897 for subdimensions.

### Ethical approval

The study has been approved by the ethical committee of the College of Nursing of Wannan Medical College (LL-2022BH02); all methods were performed in accordance with the Helsinki guidelines [41].

### Statistical analysis

Statistical analysis was conducted utilizing SPSS (Statistical Product and Service Solutions, version 23). Frequencies, percentages, means, and standard deviations were used to describe the distribution of participants' sociodemographic data, job stress, practice environment, and patient safety attitudes. The Shapiro–Wilk test was used to assess the normal distribution of variables. As the variables were skewed distribution, the Spearman correlation coefficient was employed to analyze correlations. To enhance the understanding of the results, we use R (version 4.3.1) to visually represent the correlations between the variable dimensions. Given a skewed distribution, Smart PLS (version 3) was preferred to analyze the structural equation model SEM and test the theoretical hypothesis model.

For assessing reflective models, factor loading (> 0.708), Cronbach's α (> 0.9), and composite reliability (CR > 0.9) were adopted [42, 43]. Convergent validity was assessed using the average variance extracted (AVE), which should be higher than 0.5, and discriminant validity was used the heterotrait-monotrait (HTMT) ratio of the correlations, which should be less than 0.85 for the conceptually different constructs [44].

The structural models and determining that the sample predictive power of the model is explained by the coefficient of determination R^2^, when the model does not have collinearity issues (variance inflation factor, VIF < 5) [45]. R^2^ ranges from 0 to 1, with higher values indicating greater explanatory power. Henseler and colleagues state that R^2^ values of 0.75, 0.50, and 0.25 can be considered significant, moderate, and weak [46]. The predictive accuracy of the model was explained using the blindfolding-based cross-validated redundancy measure Q^2^, which should have a value greater than 0 to indicate the predictive accuracy of this structural equation model. As a rule of thumb, Q^2^ values above 0, 0.25, and 0.50 indicate small, medium, and large predictive accuracies for PLS pathway models [42]. The global fit of the PLS modeling was explained using the goodness-of-fit (GoF), $\mathrm{GoF}=\sqrt{\overline{\mathrm{Communality}\ast\overline{\mathrm R^2}}}$, with 0.1, 0.25, and 0.36 being the small, medium, and large values of the global fit of the model, respectively [47].

Finally, we used the bootstrapping method with 5,000 resamples to evaluate the significance of the path coefficient and mediation effect (t-values should be higher than 1.96 or smaller than -1.96 for the two-tailed test).

## Results

### Participants' characteristics

Three hundred and seventy participants' socio-demographic characteristics are shown in Table 1. The average age of the new nurses was 25.43 ± 1.56 years. The majority (61.6%) was 23 ~ 26 years old, female (92.7%), 63.5% possessed advanced diploma. About one third (31.6%) had less than 6 months of work experience, and 33.2% had more than 18 months. Majority of them (40.5%)worked in internal medicine.

**Table 1:** Socio-demographic characteristics of the sample (*N* = 370)

Variables	n	%
Age (years)		
21 ~ 23	41	11.1
23 ~ 26	228	61.6
27 ~ 29	101	27.3
Gender		
Male	27	7.3
Female	343	92.7
Education level		
Advanced diploma	210	56.8
Bachelor or higher	160	43.2
Months of Work		
< 6	117	31.6
6 ~ 12	59	15.9
12 ~ 18	71	19.1
> 18	123	33.2
Work of units		
Medical	150	40.5
Surgical	70	18.9
Gynecology/Pediatric	40	10.8
Operating room	11	3.0
Emergency room/Intensive care unit	82	22.2
Outpatient department	17	4.6
Locale		
Wuwei	36	9.7
Tongling	30	8.1
Chuzhou	40	10.8
Huaibei	34	9.2
Anqing	96	25.9
Wuhu	134	36.2

### Descriptive statistics

Descriptive statistics of practice environment, job stress and patient safety attitudes are shown in Table 2. The skewness and kurtosis of the three scores ranged from -0.582 ~ 0.770 and -0.259 ~ 1.471 respectively, combined with the results of the Shapiro–Wilk test, *p* values were all < 0.001. It was concluded that the scale scores did not follow a normal distribution.

**Table 2:** Descriptive statistics for the practice environment, job stress and patient safety attitudes

Variables	Number of Items	Mean	SD	Cronbach's α
Practice Environment^a^	31	100.25	13.18	0.970
Nurse Participation in Hospital Affairs	9	28.31	4.29	0.909
Nursing Foundations for Quality of Care	10	33.01	4.15	0.927
Nurse Manager Ability, Leadership, and Support of Nurses	5	16.34	2.34	0.833
Staffing and Resource Adequacy	4	12.67	2.08	0.848
Collegial Nurse–Physician Relations	3	9.91	1.40	0.822
Job Stress^a^	35	68.92	16.76	0.961
Nurse Profession and Work	7	15.27	3.94	0.844
Workload and Time Allocation	5	10.65	3.39	0.885
Work Environment and Resources	3	5.54	2.08	0.847
Patient Care	11	23.04	5.24	0.900
Management and Dressing	9	14.44	5.15	0.944
Patient Safety Attitudes^b^	31	126.99	14.39	0.911
Teamwork Climate	6	24.75	3.35	0.682
Safety Climate	7	28.46	4.11	0.763
Perceptions of Management	4	16.98	2.58	0.753
Job Satisfaction	5	21.02	3.31	0.897
Working Conditions	5	21.06	3.11	0.863
Stress Recognition	4	14.72	3.73	0.872

*SD* standard deviation

^a^4-point scale with a range of 1–4

^b^5-point scale with a range of 1–5

The average score of practice environment was 100.25 ± 13.18, and the mean score for job stress was 68.92 ± 16.76. The average patient safety attitudes score was 126.99 ± 14.39, indicating that the participants' patient safety attitudes score was at a moderate level, and the 'perceptions of management' was highest. The Cronbach's α of the three variables ranged from 0.911 to 0.970, and the Cronbach's α of each dimension ranged from 0.682 to 0.944, indicating that these scales have good reliability in this study.

### Correlation of variables

Table 3 shows the results of the Spearman correlation analysis between the main variables. There was a positive correlation between practice environment and patient safety attitudes (*r* = 0.660, *p* < 0.001). Job stress was significantly negatively associated with both the practice environment (*r* = -0.346, *p* < 0.001) and patient safety attitudes (*r* = -0.388, *p* < 0.001).

**Table 3:** Correlation coefficients of variables

Variables	1	2	3
1. Practice Environment	1	–	–
2. Job Stress	-0.388**	1	–
3. Patient Safety Attitudes	0.660**	-0.346**	1

** p < 0.001

**Fig. 2:**
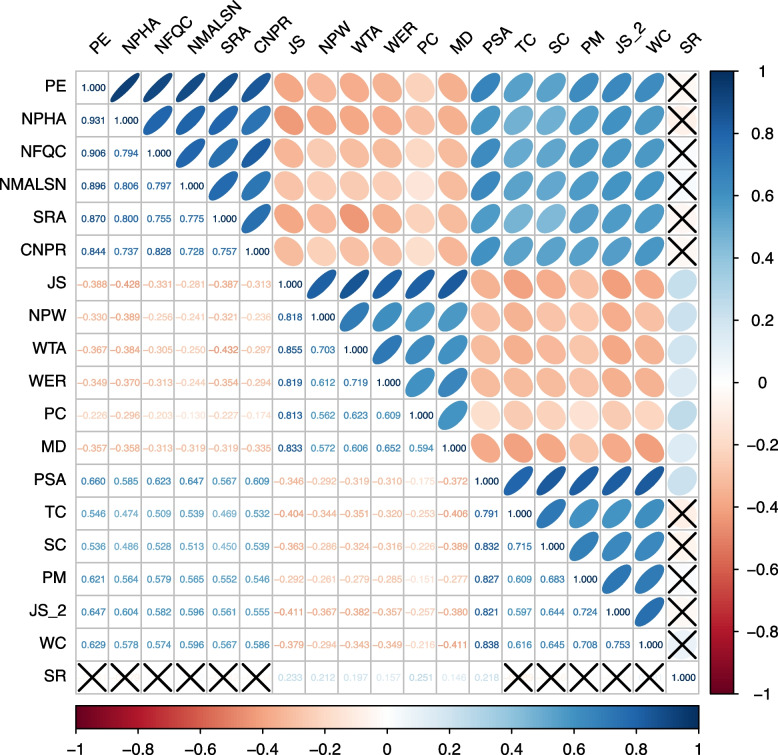
Correlations between practice environment, job stress, and patient safety attitudes. *Note*: blue, positive correlation; red, negative correlation; dark color, high correlation; upper half, color plots; lower half, numerical plots; × , *p* > 0.05; PE, practice environment; NPHA, nurse participation in hospital affairs; NFQC, nursing foundations for quality of care; NMALSN, nurse manager ability, leadership, and support of nurses; SRA, staffing and resource adequacy; CNPR, collegial nurse-physician relations; JS, job stress; NPW, nurse profession and work; WTA, workload and time allocation; WER, work environment and resources; PC, patient care; MD, management and dressing; PSA, patient safety attitudes; TC, teamwork climate; SC, safety climate; PM, perceptions of management; JS_2, job satisfaction; WC, working conditions; SR, stress recognition

The detailed results of the correlation between the variables and their dimensions are depicted in Fig. 2. The results were much the same as the relationship between the major variables. The stress recognition dimension of the patient safety attitudes scale has a significant positive correlation with the dimensions of job stress and has no significant correlation with the dimensions of practice environment and patient safety attitudes.

### Test of the hypothesized model

#### Reliability and validity of the measures

**Fig. 3:**
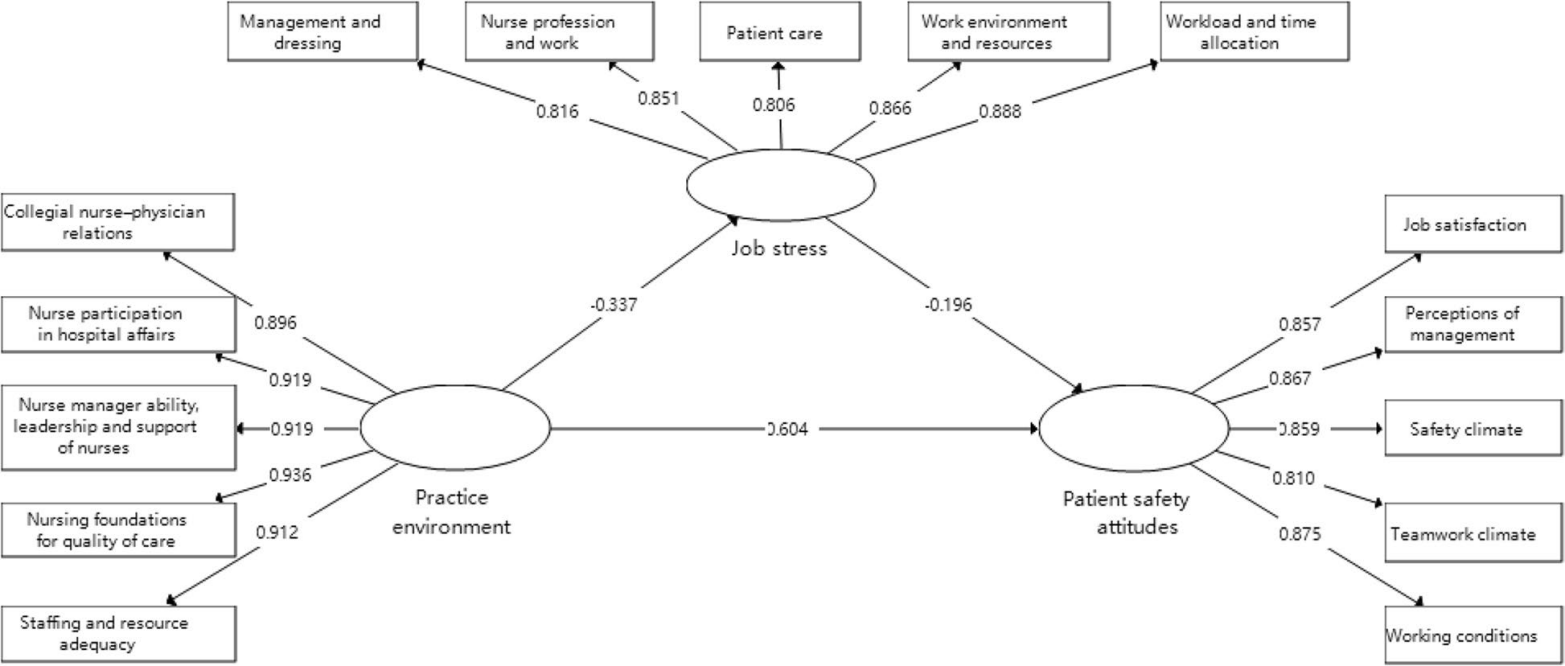
Structural equation modeling of practice environment, job stress and patient safety attitudes

The SEM was carried out using the PLS method of Smart PLS 3 (Fig. 3).

Factor loading, Cronbach's α, and CR coefficients were used in the reflective measurement model to assess the item reliability and internal consistency reliability of the model. AVEs were used to evaluate the convergent validity of the model, as shown in Table 4. Except for the excluded variable 'stress recognition' (factor loading = -0.091), all factor loadings were greater than 0.708, indicating that the model had acceptable item reliability. All Cronbach's α and CR coefficients were above 0.9, indicating acceptable consistency reliability. The Cronbach's α and CR coefficients for the practice environment variable were above 0.95, suggesting that there may be redundant items within this variable [43]. All AVEs were greater than 0.5 and the results indicated that the model had acceptable convergent validity.

**Table 4:** Reliability & convergent validity of the model

Variables	Factor loadings	α	CR	AVE
Practice Environment		0.953	0.963	0.840
Nurse Participation in Hospital Affairs	0.919			
Nursing Foundations for Quality of Care	0.912			
Nurse Manager Ability, Leadership, and Support of Nurses	0.919			
Staffing and Resource Adequacy	0.936			
Collegial Nurse–Physician Relations	0.896			
Job Stress		0.902	0.926	0.716
Nurse Profession and Work	0.851			
Workload and Time Allocation	0.888			
Work Environment and Resources	0.866			
Patient Care	0.806			
Management and Dressing	0.816			
Patient Safety Attitudes		0.909	0.931	0.729
Teamwork Climate	0.857			
Safety Climate	0.867			
Perceptions of Management	0.859			
Job Satisfaction	0.810			
Working Conditions	0.875			

α, Cronbach's α; *CR* Composite reliability, *AVE* Average variance extracted

The discriminant validity of the model was assessed using the HTMT ratios of the correlations, as shown in Table 5. The variables fall under different conceptual constructs. When the HTMT < 0.85, it indicated that the discriminant validity of the model was acceptable [44]. Therefore, the measurement model used in this study demonstrated acceptable reliability and validity.

**Table 5:** Discriminant validity of the model

Variables	1	2	3
1. Practice Environment	–	–	–
2. Job Stress	0.349	–	–
3. Patient Safety Attitudes	0.720	0.428	–

#### Assessing structural model

Table 6 demonstrates the goodness of fit for the SEM. The variables in this SEM had VIF values ranging between 2.024 and 4.371, indicating there were no collinearity issues [45]. The explanatory power within the model was represented by the coefficient of determination R^2^. The results suggested that the model could account for 11.3% of practice environment and 48.3% of patient safety attitudes, measured as weak and moderate levels of explanatory power [46], respectively.

**Table 6:** Goodness of fit of the structural model

Variables	SSO	SSE (Overlapping)	SSE (Communality)	Q^2^ (Redundancy)	Q^2^ (Communality)	R^2^
Practice Environment	1850	1850	468.745		0.747	
Job Stress	1850	1710.885	799.046	0.075	0.568	0.113
Patient Safety Attitudes	1850	1205.530	764.140	0.348	0.587	0.483

*SSO* Standardized Solution Output, *SSE* Standardized Structural Equation Residuals; Q^2^ = 1-SSE/SSO; Q^2^(Redundancy) = 1-SSE(Overlapping)/SSO; Q^2^ = (Communality) = 1-SSE(Communality)/SSO

The blindfold-based cross-validation redundancy measure Q^2^ denotes the precision of the structural model forecasts. The results showed that the model had a low predictive accuracy for the practice environment (Q^2^ = 0.075) and a medium predictive accuracy for patient safety attitudes (Q^2^ = 0.348) [42]. The GoF considers structural and measurement models and tests their quality simultaneously. The GoF value was estimated at 0.435, indicating that the model fits well [47].

#### Hypothetical test

The conceptual model was analyzed using PLS-SEM according to the bootstrapping approach in Smart PLS 3. There was a significant effect of practice environment on job stress (β = -0.337, t = 6.120) and patient safety attitudes (β = 0.604, t = 13.766), indicating that the better the practice environment, the lower the job stress, and the more positive the patient safety attitudes. There was also a significant effect of job stress on patient safety attitudes (β = -0.196, t = 4.677), indicating that the higher the job stress of the new nurses, the worse the patient safety attitudes (Table 7).

**Table 7:** Summary of hypotheses testing results

Path	Standard path coefficients	t-value	Results	95%CI
Practice Environment—> Job Stress	-0.337	6.120	Supported	(-0.445, -0.233)
Job Stress—> Patient Safety Attitudes	-0.196	4.677	Supported	(-0.280, -0.116)
Practice Environment—> Patient Safety Attitudes	0.604	13.766	Supported	(0.516, 0.688)

#### Indirect effects

The mediating role of job stress in the relationship between practice environment and patient safety attitudes is shown in Table 8, which confirmed the indirect effect of practice environment on patient safety attitudes through job stress (β = 0.066, t = 3.690), showing the mediating effect of job stress. At the same time, the total effect of the practice environment on patient safety attitudes(β = 0.670, t = 17.811) was derived, with the mediating effect accounting for 9.9% of the total effect.

**Table 8:** Summary of mediation effect test results

Effect	Path	Standard path coefficients	t-value
Indirect effect	Practice Environment—> Job Stress—> Patient Safety Attitudes	0.066	3.690
Total effect	Practice Environment—> Patient Safety Attitudes	0.670	17.811

## Discussion

The study provided empirical support for the JD-R model using new nurses' patient safety attitudes as an organizational outcome and investigated how practice environment and job stress affect these attitudes. Overall, our study confirms the previous hypothesized model.

### New nurses' patient safety attitudes

The results of our study revealed that the level of patient safety attitudes of new nurses was at a moderate level. The findings are similar to those of Li YZ et al. [48], Zhang, F et al. [49], and Ünver S, et al. [50]. These outcomes show that new nurses generally have more positive attitudes towards patient safety. Among the six dimensions, the findings showed that the highest mean score was for "perceptions of management", while the lowest score was for "stress recognition". This finding is inconsistent with that of Zhang, F et al. [49], and Ünver S, et al. [50]. Their studies reported that the highest score dimension for patient safety attitudes was the teamwork climate. Some scholars consider teamwork to be an essential factor in improving patient safety, and nurses with positive attitudes towards patient safety were more likely to cooperate and contribute to teamwork [51]. The reason for this inconsistency could be the differences in the study populations. Our study participants are new nurses undergoing standardized training and rotating between different departments in the hospital. In this case, they require consistent management and training. Therefore, perceptions of management are extremely important in shaping new nurses' attitudes towards patient safety. When management prioritizes patient safety, it encourages new nurses to enhance their attitudes toward patient safety. However, a couple of studies [52–54] have shown that the stress recognition dimension received the lowest score. This suggests that new nurses may have difficulty identifying stressful situations they come across in the workplace. The reason for this may be that usually, new nurses are assigned to do the relatively easy caring work, but not to care for critically ill patients. Besides, there are usually mechanisms set up in the unit to deal with emergencies, so they believe that even in high-pressure situations, working performance is not significantly affected [50]. Based on this finding, it is recommended that clinical managers should act as mentors to guide new nurses in identifying and managing the stresses they encounter.

### Relationship between practice environment, job stress, and patient safety attitudes

This study also found that new nurses' patient safety attitudes were positively correlated with their practice environment and were negatively correlated with their job stress, which is consistent with the findings of existing studies [55–58]. It is worth noting that the stress perception dimension of the safety attitudes questionnaire was positively correlated with job stress and not significantly correlated with practice environment. This result is unusual because it differs from the other dimensions of the Safety Attitudes Questionnaire, which may be due to the fact that when job stress increases, new nurses have to expend more energy dealing with these stressors, which tends to produce higher perceptions of stress.

In our study, the practice environment had a direct positive effect on patient safety attitudes, which is similar to that of Al Ma'mari Q et al. [55]. They also found that the practice environment predicted nurses' patient safety attitudes and that there was a relationship between a good work environment and the quality of nursing care and improved patient safety outcomes [58]. We concluded that a positive nursing practice environment promotes, among other factors, the involvement of new nurses in quality management, improved collaboration between healthcare providers and the provision of adequate resources. These conditions can be used as working resources to improve attitudes to patient safety and thus provide safer care for patients. It suggests that nurse leadership is a key factor in building and sustaining a healthy work environment. Effective nurse leadership is a precursor to a healthy work environment [59]. Therefore, nurse managers should understand and improve the status quo of the clinical practice environment in different departments and improve their nursing leadership to promote safe care.

Our results indicated that job stress had a direct negative effect on new nurses' patient safety attitudes, which congruent with the findings of Yalçın Akgül G et al. [56], whose studies found that a statistically significant negative correlation between organizational stress and patient safety attitudes. Job stress is a major cause impacting the physical and mental health of healthcare workers as well as in the quality of health services [60], and working in high-pressure environments can increase the risk of care errors and adverse events [61]. Therefore, job stress is one of obstacles in shaping patient safety attitudes, and nurse managers need to identify new nurses' job stress in a timely manner and provide effective stress management interventions to reduce new nurses' job stress and promote a safe nursing environment.

### The mediating role of job stress in the relationship between practice environment and patient safety attitudes

Our findings also support the hypothesis of the practice environment has an indirect positive effect on patient safety attitudes through mediation of job stress. Several studies [55, 62, 63] have shown that many factors, such as burnout, fatigue, workload, working conditions and workplace violence, affect nurses' job stress and reduce their attitudes to patient safety. These factors can lead to adverse events by increasing nurses' stress, and with job stress serving as a mediating factor. The study's results demonstrated that the practice environment indirectly affects the safety attitudes of new nurses by reducing job stress. According to the stress and coping theory [64], stress arises from the interplay between an individual and their external environment when the individual's coping resources are insufficient to manage external environmental threats. Therefore, improving the practice environment of new nurses can help to reduce their job stress and thus improve patient safety attitudes.

### Strengths and limitations

The present study has several strengths and limitations. The first strength is that it employed the JD-R model to examine how practice environment and job stress to the patient safety attitudes of new nurses. The second strength is multicentered study with a variety of samples' characteristics, which makes the results more representative and applicability, and the third strength is the model was tested using scientific methods to assess model fit. The study's limitations include: (1) the sample size was satisfied for testing SEM based on experts' recommendation [31], but 370 samples were not large enough to make the study results more applicable to other regions or countries. (2) Some other variables within the JD-R model had not been included in this study, which may influence the comprehensiveness of J-DR model regarding new nurses' practice environment, job stress, and patient safety attitudes, and (3) we did not consider the effect of covariates on SEM, so the effect sizes in the results should be referred with caution.

## Conclusions

Patient safety attitudes are important for improving patient satisfaction and clinical outcomes in the workforce. Our study found that new nurses had moderate levels of overall patient safety attitudes. The highest score was noted for "perceptions of management" and the lowest was found for "stress recognition". It is recommended that the effective strategies be developed for new nurses to improve their patient safety attitudes further. Based on the JD-R model, to explore theoretical models of practice environment, job stress, and patient safety attitudes among new nurses and tested the fit of the theoretical model. The findings confirmed that the model had a good fit and can effectively account for the relationship between these variables. Furthermore, the findings reveal that not only do the practice environment and job stress directly affect the patient safety attitudes among new nurses, but improving the practice environment can reduce job stress and thus indirectly improve the patient safety attitudes also. As a result, job stress plays a mediating role in the relationship between practice environment and patient safety attitudes. The practical suggestions are (1) policymakers and managers should pay more attention to the practice environment and job stress of new nurses, (2) strategically enhancing the practice environment and mitigating job stress by acknowledging the variances in the practice environment across various departments, and (3) safety training should be provided to new nurses to improve their safety attitudes.

## Data Availability

The datasets used in this study are confidential but available upon request from the corresponding author.

## Abbreviations

WHO: World Health Organization
JD-R: Job Demands-Resources
SEM: Structural Equation Modeling
STROBE: Strengthening the Reporting of Observational Studies in Epidemiology
PES: Practice Environment Scale
NJSS: Nurse Job Stressors Scale
SAQ: Safety Attitudes Questionnaire
PLS: Partial Least Squares
CR: Composite Reliability
AVE: Average Variance Extracted
HTMT: Heterotrait-Monotrait
GoF: Goodness-of-Fit
SD: Standard Deviation
SSO: Standardized Solution Output
SSE: Standardized Structural Equation Residuals
